# Sagittal Split Ramus Osteotomy in the Shortest Buccal Bone Marrow Distances of the Mandible on the Coronal Plane

**DOI:** 10.1155/2021/5586498

**Published:** 2021-03-18

**Authors:** Chun-Ming Chen, Han-Jen Hsu, Ping-Ho Chen, Shih-Wei Liang, I-Ling Lin, Kun-Jung Hsu

**Affiliations:** ^1^School of Dentistry, College of Dental Medicine, Kaohsiung Medical University, Kaohsiung, Taiwan; ^2^Department of Oral and Maxillofacial Surgery, Kaohsiung Medical University Hospital, Kaohsiung Medical University, Taiwan; ^3^Department of Medical Laboratory Science and Biotechnology, College of Health Sciences, Kaohsiung Medical University, Kaohsiung, Taiwan; ^4^Department of Dentistry, Kaohsiung Municipal Ta-Tung Hospital, Kaohsiung, Taiwan

## Abstract

**Purpose:**

This study investigated the relationship between the shortest buccal bone marrow of the ramus and skeletal patterns.

**Materials and Methods:**

Using cone-beam computed tomography data (specifically, the A point-nasion-B point (ANB) angle), we divided patients into three groups as follows: skeletal class I (0° < ANB < 4°), class II (ANB: ≥4°), and class III (ANB: ≤0°). Sixteen vertical sections in the coronal plane were taken starting from slice 0 (original intact mandibular canal) anteriorly at 2 mm intervals to slice 15 (30 mm). The thickness of the mandible (M) and shortest buccal bone marrow (SBM) were measured. The data of SBM were divided into two groups (SBM ≥ 1 mm and SBM < 1 mm). For each skeletal pattern, an SBM value < 1 mm was considered to indicate a high possibility of postoperative nerve paresthesia and bad split.

**Results:**

The three skeletal pattern groups also did not significantly differ in their M values for all sections. The mean SBM values of class III (0.91–2.11 mm) at 6–16 mm anterior to the mandibular foramen were significantly smaller than those of class II (1.53–3.17 mm). Comparing the occurrence ratio of SBM < 1 mm, the highest and lowest probabilities in class III (55% and 21.7%, respectively) were significantly larger at 6–20 mm anterior to the mandibular foramen than those in class II (28.3% and 5%, respectively).

**Conclusion:**

Class III had a significantly shorter SBM distance and higher SBM occurrence probability than class II at the mandibular ramus region, implying that class III participants are more likely than class II participants to have nerve paresthesia and bad split after sagittal split ramus osteotomy.

## 1. Introduction

Malocclusion is frequently accompanied by facial deformities, which considerably affects not only respiration, eating, and speech but also social interaction. Facial deformities have a significant negative effect on perceptions of social functionality, including employability, honesty, and trustworthiness [1]. Sagittal split ramus osteotomy (SSRO) is commonly used to correct mandibular protrusion, retrusion, and asymmetry. Its advantages include enhanced bone healing because of larger overlapping segments, better and easier postoperative airway management, immediate postoperative jaw mobilization, leading to better oral hygiene and quicker improvement to a regular diet, and better speech and social activity [2]. The major complications of SSRO include inferior alveolar nerve injury, resulting in lower lip paresthesia; a bad or unfavorable split, leading to bony malunion; and unpredictable condylar position, leading to an undesired postsurgical shift in the occlusion.

The sagittal osteotomy line of SSRO starts at the buccal cortex of the mandibular ramus and body. The osteotomy incision is made through the cortex into the buccal bone marrow, and the osteotomes are then inserted into the marrow to engage the lower border of the mandible, followed by mandibular splitting. Many researchers [3–6] have reported the postoperative complications of SSRO, especially inferior alveolar nerve damage and poor mandibular split. However, the shortest buccal bone marrow (SBM) distance of the mandible is the most critical risk factor contributing to inferior alveolar nerve injury and poor or unfavorable split. No study has focused on the relationship between SBM distance and skeletal patterns (classes I, II, and III). Therefore, we investigated whether the three skeletal pattern groups differed in SBM values and speculated the occurrence possibility of SBM < 1 mm in the different skeletal patterns.

## 2. Material and Methods

In this study, we collected the cone-beam computed tomography data of 90 participants at the Department of Dentistry, Kaohsiung Medical University Hospital, Kaohsiung, Taiwan. NNT viewer software was used to view the captured images. Ninety participants were divided into three skeletal classes (I, II, and III) according to their A point-nasion-B point (ANB) angle, with 30 participants per class. Participants with symptoms such as craniofacial injury, tumors, and congenital craniofacial deformities were excluded. The reference plane of the three-dimensional image was the FH plane (horizontal plane), which is defined as the plane constituted by the three points that pass through the right orbitale and bilateral porion.

**Figure 1:**
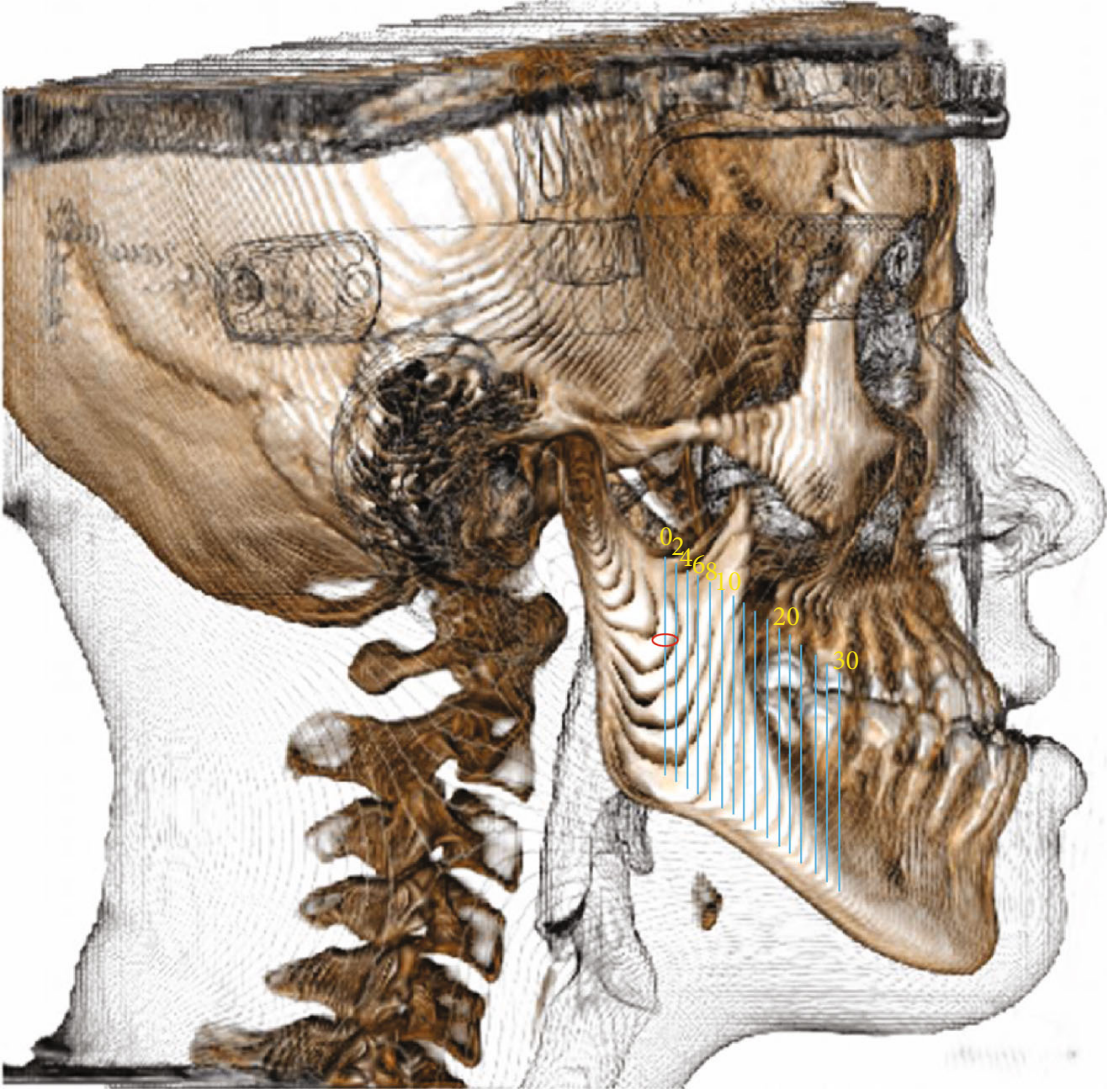
Red circle: mandibular canal (base plane: 0 mm). Sixteen vertical slices (blue lines) from 0 mm forward to 30 mm.

**Figure 2:**
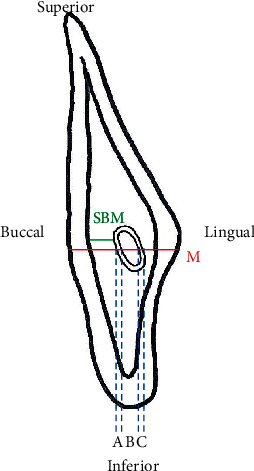
Red line distance (M: thickness of mandible). (a) Buccal cortex of the mandibular canal sheath. (b) Dimension of the mandibular canal. (c) Lingual cortex of the mandibular canal sheath. Green line distance (SBM: shortest buccal bone marrow distance).

Section 0 was set as the vertical section in the coronal plane that enables observation of the complete mandibular foramen anteriorly from the posterior border of the ramus (Figure 1). The section 2 mm anterior to section 0 was considered to be section 1, the section 2 mm anterior to that was considered section 2, and so on until a position 30 mm anterior to the start of section 0. Thus, 16 sections were demarcated in total. In clinical observation, section 16 (at 30 mm) includes the vertical osteotomy line of SSRO at the second and first molar areas. We defined a horizontal line segment (M) that passes through the center of the inferior alveolar nerve; M starts from the buccal side of the mandible and ends at the lingual cortical bone (Figure 2). Landmarks on M were then identified, and the following line segments were defined: buccal cortex of the mandibular canal sheath (A), dimension of the mandibular canal (B), and lingual cortex of the mandibular canal sheath (C). We then measured the SBM located between the inner side of the mandibular buccal cortical bone and the buccal side of the mandibular canal sheath. The data of SBM were divided into two groups (SBM ≥ 1 mm and SBM < 1 mm).

All statistical analyses were conducted using IBM SPSS 20 (SPSS Inc., Chicago, IL, USA). Analysis of variance was used to examine the differences between the three skeletal pattern groups, with the Tukey *post hoc* analysis. For each skeletal pattern, an SBM value < 1 mm was considered to indicate a high possibility of postoperative nerve paresthesia and poor split. A chi-square test was used to examine the intergroup differences, with the Bonferroni *post hoc* analysis. A Wilcoxon signed-rank test was used to examine, for all three groups, the left- and right-side measurement values.

The null hypothesis was that there is no significant difference in the occurrence probability of SBM < 1 mm among the skeletal patterns. This retrospective study was approved by the clinical trial committee of Kaohsiung Medical University Hospital (IRB No. KMUH-IRB-20160066).

## 3. Results

The 90 participants included 30 men and 60 women. Of them, 30 participants (9 men and 21 women) were in the skeletal class I group (mean age, 25.2 years; ANB angle, 1.73°), 30 (6 men and 24 women) were in the skeletal class II group (mean age, 27.8 years; average ANB angle, 7.1°), and 30 (15 men and 15 women) were in the skeletal class III group (mean age, 22.8 years; average ANB angle, −4.1°).

**Figure 3:**
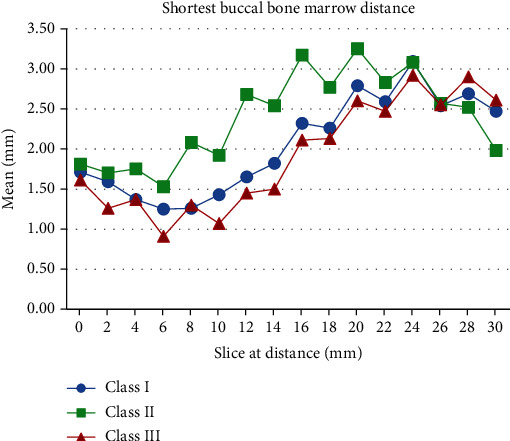
The shortest buccal bone marrow (SBM) distances in the three skeletal patterns.

Analysis of variance was used to determine if the skeletal pattern groups differed in their anatomical structures in the buccal-lingual direction of the mandibular canal. In Table 1, the buccal cortex of the mandibular canal sheath (A) and the lingual cortex of the mandibular canal sheath (C) were <1 mm. In the same section, A was always larger than C within a skeletal class. The dimensions of the mandibular canal (B) were similar in the three skeletal patterns. The mean B value ranged from 2.15 mm to 2.86 mm. We found that the dimensions of the mandibular canal and sheath (A + B + C) were approximately 3.5–5 mm. In Table 2, the M value was the mandibular thickness; for most sections, the mean M value was 10–13 mm. The three skeletal pattern groups also did not significantly differ in their M values for all sections. The mean SBM values of class III (0.91–2.11 mm) at 6–16 mm anterior to the mandibular foramen were significantly smaller than those of class II (1.53–3.17 mm; Figure 3).

A Wilcoxon signed-rank test was used to examine, for all three groups, whether the left- and right-side measurement values significantly differed (Table 3). Relative to the left-side measurement values, the right-side measurement values were significantly larger only for class I patients in section 10 (20 mm) and class II patients in section 14 (28 mm). The rate of occurrence of SBM < 1 mm was significantly higher in class III participants at 6–20 mm anterior to the mandibular foramen than in class II participants (Table 4). Therefore, the null hypothesis was rejected. The highest and lowest SBM probabilities at all sections were 55% and 21.70%, respectively, for the class III group and 28.30% and 5.00%, respectively, for the class II group.

## 4. Discussion

Mandibular deformities can be categorized as either deficiency or protrusion. SSROs are frequently used to correct both types of mandibular deformities through mandibular advancement or setback, respectively. The mandibular canal contains the inferior alveolar neurovascular bundle, which is the most critical anatomical structure for SSRO. Pogrel et al. [7] reported that the inferior alveolar vein lies superior to the nerve and the artery lies on the lingual side of the nerve. Ozturk et al. [8] indicated that most mandibular canals are either in contact with or close to the lingual cortical plate in the molar region, which is consistent with our findings. From section 11 to 16 (third to the first mandibular molar region), the inferior alveolar canal was close to the mandibular lingual cortical plate. Using magnetic resonance imaging, Ikeda et al. [9] reported that the greater diameter of the mandibular canal with the bony cortex averaged 4.1 mm near the mandibular foramen and 3.4 mm in the middle of the canal; these findings are similar to our results (A + B + C = 3.5–5 mm). In our study, mandibular width (M value) was not significantly different among the three skeletal patterns. Therefore, the diameter of the mandibular canal and its surrounding cortex could be consistent with real expectations.

The SBM is a critical anatomical location when considering the safety of SSROs. Damage to the inferior alveolar nerve can be caused not only by actual contact with the surgical drills but also by excessive drilling pressure, mechanical stress, and thermal effects. Marenzi et al. [10] reported that the surface micromorphology of the bone drill bur, which influences the contact area between the drill bur and bone, can contribute to thermal necrosis of bone. These aspects can cause permanent or transient sensory alterations. Shirota et al. [11] investigated the effectiveness of piezoelectric and conventional bur methods in reducing surgical complications after SSRO for mandibular setback. They reported that piezoelectric surgery reduced neither blood loss nor the incidence of neurosensory disturbance in SSRO. By contrast, Kokuryo et al. [12] stated that ultrasonic surgery may be more effective than conventional surgery to reduce the incidence of neurosensory disturbance after SSRO and promote recovery from neurosensory disturbance.

The SBM thickness and split techniques play crucial roles in preventing nerve damage during surgery treatment. If the SBM value is too small for osteotome manipulation, the osteotome may split directly toward the inferior alveolar neurovascular bundle, damage it, and result in lower lip paresthesia. Moreover, an unanticipated osteotome run laterally or medially may lead to unfavorable splits. Möhlhenrich et al. [13] evaluated the lingual fracture patterns after SSRO with Hunsuck–Epker modifications by an additional inferior border osteotomy using a bur or ultrasonic device. They observed no relationship between the split technique and the fracture pattern. The bone cut on the inferior border did not improve split control but rather increased the risk of unwanted fractures and extended the operation time. Furthermore, the SSRO technique relies on a directed fracture involving bone thickness, bone density, and various SSRO-related biomechanical properties. Rougier et al. [14] measured the hardness of the ramus and conducted traction-to-fracture tests. They recommended using a Lindemann bur rather than a reciprocating saw for the corticotomy and opting for a wide approach to the medullary bone to easily introduce the osteotome, thereby reducing the risk of a poor split. Therefore, correct 3D presurgical planning is required to avoid damage to the mandibular canal.

During SSRO, McLeod and Bowe [15] concluded that the inferior alveolar nerve may be damaged by traction during stripping of the soft tissue of the medial ramus, incorrect medial bone cut, unfavorable split, improper retraction or advancement of the distal segment, misjudged placement of a miniscrew, and compression from the proximal segment and distal segment fixation. Zaroni et al. [16] explored the postoperative complications of orthognathic surgery and reported a 19.2% complication rate, including postoperative malocclusion, hemorrhage, inferior alveolar nerve injury, bad split, and infection. Gennaro et al. [17] investigated the intraoperative frequency of nerve exposure and reported a high incidence of neurosensory disturbance in the lower lip and chin after SSRO. Politis et al. [18] stated that postoperative changes in lower lip sensation were 15.1% after SSRO.

Steenen and Becking [19] reviewed unfavorable and unanticipated splits during SSRO and reported a 2.3% incidence of poor split. They also observed that the buccal plate of the proximal segment and the lingual plate of the distal segment were frequently encountered fracture patterns. Studies have indicated that the buccal plate is more prone to result in bad split than the lingual plate. In the process of splitting the mandible into the mesial and distal portions using SSRO, an excessively small mandibular SBM may cause the inferior alveolar nerve to be injured when operating surgical instruments and increase the probability of unexpected bone fracture. Therefore, SBM is the most critical risk factor leading to inferior alveolar nerve injury and bad split during SSRO.

Although the mean values between skeletal pattern groups are essential in determining whether anatomical structures increase the likelihood of postoperative complications, the probability for the value to be minimal is more strongly correlated with the occurrence of postoperative complications (nerve paresthesia or bad split) than the mean values are. Huang et al. [20] reported that the measurement values were significantly smaller for participants with nerve paresthesia than for those without at the 16, 18, 20, or 24 mm slices anterior to the mandibular foramen. Many studies [21–25] on SBM values have been conducted. Yamamoto et al. [21] noted a significant difference among skeletal class III participants who had undergone SSRO surgery and found that nerve paresthesia occurred in 0% of participants with SBM ≥ 1 mm and in 61.5% of participants with SBM < 1 mm. Yamauchi et al. [23] observed significant postoperative nerve paresthesia in 57.1% of participants with SBM < 1 mm and in 7.7% of participants with SBM > 2 mm. However, whether the SBM value is significantly different between classes II and III remains unclear. Our findings indicate that in sections 3–8 (6–16 mm anterior to the mandibular foramen), the mean SBM values were significantly larger in class II patients (1.53–3.17 mm) than in class III patients (0.91–2.11 mm). This indicates that the occurrence of postoperative complications differs between class II and class III patients after SSRO.

Concerning postoperative complications, the occurrence probability rate of SBM was as essential as the size of SBM. We used a chi-square test to examine the probability of having minimal SBM values and demonstrated that for many of the sections within the region where SSRO is conducted, the probability rate of having a minimal SBM value was larger for class III participants than for class II participants. For the eight sections (sections 3–10) that were 6–20 mm anterior to the mandibular foramen, this probability was significantly larger for the class III group than for the class II group, with the differences in probability between the two groups being 16.70% to 26.60% for all sections.

Taking our findings and the results of Yamamoto et al. [21] and Yamauchi et al. [23] together, we conclude that class III participants are more likely to have post-SSRO nerve paresthesia than skeletal class II participants are. In this study, we examined SBM minimum values by combining the left- and right-side values to eliminate the possibility of existing differences between the left and right sides affecting the study results. Only two sections had significant differences between the left and right sides. Therefore, we conclude that the left and right sides do not significantly differ, and the left- and right-side values can be combined when conducting a statistical analysis.

## 5. Conclusion

Our data indicate that skeletal class III participants had a significantly smaller SBM than skeletal class II participants did. Furthermore, the probability of having a minimum SBM (<1 mm) was significantly higher for class III participants than for class I and II participants. Therefore, class III participants are more likely to have post-SSRO nerve paresthesia than class II participants are. Further research is required to directly determine whether the probability of having nerve paresthesia after SSRO significantly differs between class II and III participants.

## Figures and Tables

**Table 1:** The thickness of the mandibular canal and its sheaths from slice 0 (0 mm) to slice 15 (30 mm) with their skeletal patterns in the one-way ANOVA with the post hoc Tukey HSD test.

Variables	A (mean ± SD, mm)	*p* value	B (mean ± SD, mm)	*p* value	C (mean ± SD, mm)	*p* value
0 mm						
Class I	0.92 ± 0.30	0.336	2.41 ± 0.51	<0.001^∗^	0.51 ± 0.26	0.062
Class II	0.85 ± 0.34		2.29 ± 0.58	Class III > I > II	0.47 ± 0.24	
Class III	0.85 ± 0.26		2.86 ± 0.73		0.41 ± 0.16	
2 mm						
Class I	0.87 ± 0.31	0.219	2.45 ± 0.60	0.001^∗^	0.61 ± 0.30	0.026^∗^
Class II	0.77 ± 0.31		2.37 ± 0.63	Class III > I > II	0.55 ± 0.29	Class I > III
Class III	0.82 ± 0.29		2.80 ± 0.67		0.47 ± 0.22	
4 mm						
Class I	0.79 ± 0.30	0.758	2.48 ± 0.58	0.247	0.62 ± 0.31	0.217
Class II	0.75 ± 0.36		2.35 ± 0.67		0.53 ± 0.28	
Class III	0.77 ± 0.33		2.55 ± 0.77		0.59 ± 0.29	
6 mm						
Class I	0.81 ± 0.37	0.863	2.30 ± 0.65	0.684	0.71 ± 0.37	0.836
Class II	0.77 ± 0.30		2.37 ± 0.60		0.67 ± 0.38	
Class III	0.79 ± 0.38		2.39 ± 0.66		0.69 ± 0.32	
8 mm						
Class I	0.76 ± 0.32	0.958	2.43 ± 0.62	0.253	0.67 ± 0.33	0.102
Class II	0.77 ± 0.34		2.36 ± 0.70		0.56 ± 0.28	
Class III	0.75 ± 0.39		2.56 ± 0.68		0.65 ± 0.27	
10 mm						
Class I	0.81 ± 0.35	0.240	2.54 ± 0.58	0.524	0.72 ± 0.35	0.652
Class II	0.75 ± 0.35		2.45 ± 0.62		0.65 ± 0.39	
Class III	0.70 ± 0.37		2.58 ± 0.69		0.69 ± 0.34	
12 mm						
Class I	0.82 ± 0.32	0.689	2.51 ± 0.69	0.037^∗^	0.68 ± 0.29	0.196
Class II	0.78 ± 0.33		2.40 ± 0.73	Class III > II	0.58 ± 0.32	
Class III	0.77 ± 0.37		2.72 ± 0.63		0.61 ± 0.29	
14 mm						
Class I	0.93 ± 0.36	0.004^∗^	2.45 ± 0.63	0.392	0.68 ± 0.36	0.355
Class II	0.76 ± 0.37	Class I > II	2.36 ± 0.66		0.63 ± 0.35	
Class III	0.72 ± 0.36	Class I > III	2.51 ± 0.56		0.59 ± 0.34	
16 mm						
Class I	0.83 ± 0.33	0.649	2.32 ± 0.56	0.452	0.62 ± 0.33	0.377
Class II	0.79 ± 0.33		2.29 ± 0.57		0.55 ± 0.29	
Class III	0.78 ± 0.31		2.42 ± 0.59		0.61 ± 0.33	
18 mm						
Class I	0.88 ± 0.36	0.238	2.41 ± 0.59	0.515	0.60 ± 0.32	0.940
Class II	0.77 ± 0.39		2.38 ± 0.74		0.59 ± 0.32	
Class III	0.78 ± 0.37		2.52 ± 0.67		0.60 ± 0.31	
20 mm						
Class I	0.76 ± 0.31	0.562	2.48 ± 0.72	0.440	0.58 ± 0.28	0.950
Class II	0.69 ± 0.35		2.36 ± 0.77		0.58 ± 0.32	
Class III	0.71 ± 0.36		2.52 ± 0.63		0.59 ± 0.34	
22 mm						
Class I	0.88 ± 0.40	0.194	2.41 ± 0.68	0.287	0.56 ± 0.28	0.600
Class II	0.76 ± 0.43		2.28 ± 0.66		0.52 ± 0.29	
Class III	0.76 ± 0.35		2.46 ± 0.63		0.57 ± 0.31	
24 mm						
Class I	0.75 ± 0.36	0.767	2.26 ± 0.46	0.009^∗^	0.57 ± 0.29	0.541
Class II	0.73 ± 0.35		2.25 ± 0.57	Class III > I	0.50 ± 0.27	
Class III	0.70 ± 0.35		2.55 ± 0.72	Class III > II	0.54 ± 0.36	
26 mm						
Class I	0.78 ± 0.33	0.208	2.44 ± 0.73	0.183	0.57 ± 0.30	0.030^∗^
Class II	0.72 ± 0.37		2.41 ± 0.71		0.51 ± 0.28	Class III > II
Class III	0.83 ± 0.35		2.22 ± 0.67		0.67 ± 0.39	
28 mm						
Class I	0.83 ± 0.35	0.119	2.17 ± 0.50	0.700	0.53 ± 0.28	0.205
Class II	0.71 ± 0.31		2.24 ± 0.66		0.63 ± 0.34	
Class III	0.79 ± 0.31		2.26 ± 0.62		0.57 ± 0.31	
30 mm						
Class I	0.72 ± 0.35	0.138	2.31 ± 0.55	0.070	0.51 ± 0.25	0.528
Class II	0.61 ± 0.33		2.50 ± 0.84		0.53 ± 0.30	
Class III	0.70 ± 0.34		2.20 ± 0.73		0.57 ± 0.39	

A: buccal cortex of the mandibular canal sheath; B: dimension of the mandibular canal; C: lingual cortex of the mandibular canal sheath. ∗: significant, *p* < 0.05.

**Table 2:** The shortest distance of buccal bone marrow and thickness of the mandible from slice 0 (0 mm) to slice 15 (30 mm) with their skeletal patterns in the one-way ANOVA with the post hoc Tukey HSD test.

Variables	SBM (mean ± SD, mm)	*p* value	M (mean ± SD, mm)	*p* value
0 mm				
Class I	1.71 ± 1.46	0.710	9.79 ± 1.61	0.641
Class II	1.81 ± 1.33		9.55 ± 1.37	
Class III	1.61 ± 1.11		9.65 ± 1.31	
2 mm				
Class I	1.59 ± 1.42	0.149	9.96 ± 1.50	0.735
Class II	1.70 ± 1.30		9.79 ± 1.38	
Class III	1.26 ± 1.07		9.78 ± 1.30	
4 mm				
Class I	1.37 ± 1.35	0.179	10.08 ± 1.60	0.480
Class II	1.75 ± 1.22		10.21 ± 1.44	
Class III	1.37 ± 1.22		9.89 ± 1.27	
6 mm				
Class I	1.25 ± 1.29	0.013^∗^	10.25 ± 1.66	0.116
Class II	1.53 ± 1.11	Class II > III	10.38 ± 1.47	
Class III	0.91 ± 1.02		9.84 ± 1.29	
8 mm				
Class I	1.26 ± 1.31	0.001^∗^	11.09 ± 1.68	0.364
Class II	2.08 ± 1.30	Class II > I	11.25 ± 1.70	
Class III	1.30 ± 1.28	Class II > III	10.84 ± 1.44	
10 mm				
Class I	1.43 ± 1.30	0.001^∗^	11.19 ± 1.78	0.140
Class II	1.92 ± 1.92	Class II > III	11.42 ± 1.64	
Class III	1.07 ± 1.19		10.81 ± 1.65	
12 mm				
Class I	1.65 ± 1.45	<0.001^∗^	12.11 ± 1.90	0.515
Class II	2.68 ± 1.59	Class II > I	12.28 ± 1.80	
Class III	1.45 ± 1.44	Class II > III	11.90 ± 1.75	
14 mm				
Class I	1.82 ± 1.49	0.001^∗^	12.12 ± 1.84	0.392
Class II	2.54 ± 1.40	Class II > I	12.15 ± 1.64	
Class III	1.50 ± 1.58	Class II > III	11.74 ± 1.89	
16 mm				
Class I	2.32 ± 1.79	0.001^∗^	12.64 ± 1.73	0.879
Class II	3.17 ± 1.48	Class II > I	12.79 ± 1.64	
Class III	2.11 ± 1.62	Class II > III	12.76 ± 1.79	
18 mm				
Class I	2.26 ± 1.55	0.088	12.61 ± 1.72	0.905
Class II	2.77 ± 1.36		12.66 ± 1.59	
Class III	2.13 ± 2.00		12.52 ± 1.99	
20 mm				
Class I	2.79 ± 1.41	0.066	13.05 ± 1.72	0.370
Class II	3.25 ± 1.29		12.86 ± 1.57	
Class III	2.6 ± 1.89		13.33 ± 2.11	
22 mm				
Class I	2.59 ± 1.45	0.429	12.68 ± 1.71	0.424
Class II	2.83 ± 1.29		12.51 ± 1.65	
Class III	2.47 ± 1.83		12.95 ± 2.12	
24 mm				
Class I	3.09 ± 1.49	0.780	13.00 ± 1.77	0.084
Class II	3.08 ± 1.39		12.63 ± 1.55	
Class III	2.92 ± 1.68		13.39 ± 2.20	
26 mm				
Class I	2.54 ± 1.34	0.995	12.49 ± 1.78	0.267
Class II	2.57 ± 1.25		12.31 ± 1.52	
Class III	2.55 ± 1.64		12.84 ± 2.07	
28 mm				
Class I	2.69 ± 1.25	0.329	12.69 ± 1.91	0.173
Class II	2.52 ± 1.28		12.46 ± 1.43	
Class III	2.90 ± 1.59		13.09 ± 2.13	
30 mm				
Class I	2.47 ± 1.20	0.018^∗^	12.23 ± 1.78	0.909
Class II	1.98 ± 1.29	Class III > II	12.32 ± 1.46	
Class III	2.61 ± 1.28		12.37 ± 1.95	

SBM: shortest distance of buccal bone marrow; M: thickness of the mandible. ∗: significant, *p* < 0.05.

**Table 3:** From slice 0 (0 mm) to slice 15 (30 mm), the shortest buccal bone marrow distances of skeletal patterns with hemiarch comparisons.

Variables	Right (mean, mm)	Left (mean, mm)	*p* value	Significant
0 mm				
Class I	1.75	1.68	0.931	
Class II	1.84	1.77	0.861	
Class III	1.4	1.82	0.107	
2 mm				
Class I	1.75	1.43	0.270	
Class II	1.84	1.56	0.317	
Class III	1.4	1.12	0.107	
4 mm				
Class I	1.37	1.38	0.648	
Class II	1.74	1.76	0.855	
Class III	1.17	1.58	0.065	
6 mm				
Class I	1.37	1.13	0.254	
Class II	1.46	1.61	0.409	
Class III	0.83	0.98	0.414	
8 mm				
Class I	1.31	1.21	0.626	
Class II	1.97	2.2	0.289	
Class III	1.17	1.42	0.276	
10 mm				
Class I	1.42	1.43	0.571	
Class II	1.92	1.92	0.838	
Class III	0.98	1.17	0.493	
12 mm				
Class I	1.67	1.63	0.577	
Class II	2.70	2.65	0.904	
Class III	1.41	1.48	0.511	
14 mm				
Class I	1.86	1.78	0.449	
Class II	2.42	2.67	0.358	
Class III	1.43	1.56	0.861	
16 mm				
Class I	2.51	2.14	0.156	
Class II	3.18	3.15	0.733	
Class III	2.05	2.16	0.675	
18 mm				
Class I	2.15	2.37	0.374	
Class II	2.69	2.84	0.495	
Class III	2.1	2.17	0.568	
20 mm				
Class I	2.97	2.61	0.044^∗^	*R* > *L*
Class II	3.42	3.08	0.175	
Class III	2.68	2.53	0.517	
22 mm				
Class I	2.55	2.64	0.556	
Class II	2.85	2.8	0.845	
Class III	2.40	2.54	0.750	
24 mm				
Class I	3.21	2.98	0.294	
Class II	3.23	2.92	0.414	
Class III	3.03	2.81	0.304	
26 mm				
Class I	2.28	2.8	0.056	
Class II	2.72	2.43	0.581	
Class III	2.57	2.53	0.665	
28 mm				
Class I	2.86	2.52	0.302	
Class II	2.83	2.27	0.017^∗^	*R* > *L*
Class III	3.06	2.74	0.116	
30 mm				
Class I	2.55	2.39	0.585	
Class II	2.05	2.03	0.962	
Class III	2.55	2.67	0.914	

∗: significant (*p* < 0.05) in the Wilcoxon signed-rank test.

**Table 4:** From slice 0 (0 mm) to slice 15 (30 mm), the percentage in the shortest buccal bone marrow distance (SBM < 1 mm) with their skeletal patterns.

Variables	Class I	Class II	Class III	Total	*p* value	Significant
0 mm	40.00%	25.00%	33.30%	32.80%	0.215	
2 mm	40.00%	28.30%	41.70%	36.70%	0.256	
4 mm	41.70%	26.70%	45.00%	37.80%	0.088	
6 mm	50.00%	28.30%	55.00%	44.40%	0.008^∗^	Class I > II, class III > II
8 mm	51.70%	20.00%	45.00%	38.90%	0.001^∗^	Class I > II, class III > II
10 mm	41.70%	21.70%	53.30%	38.90%	0.002^∗^	Class III > II
12 mm	41.70%	16.70%	41.70%	33.30%	0.004^∗^	Class I > II, class III > II
14 mm	33.30%	11.70%	38.30%	27.80%	0.002^∗^	Class I > II, class III > II
16 mm	21.70%	5.00%	23.30%	16.70%	0.012^∗^	Class I > II, class III > II
18 mm	23.30%	8.30%	30.00%	20.60%	0.011^∗^	Class III > II
20 mm	6.70%	5.00%	21.70%	11.10%	0.006^∗^	Class III > II
22 mm	11.70%	8.30%	21.70%	13.90%	0.089	
24 mm	3.30%	5.00%	10.00%	6.10%	0.284	
26 mm	8.30%	8.50%	20.00%	12.30%	0.083	
28 mm	5.00%	10.20%	11.70%	8.90%	0.406	
30 mm	5.00%	24.10%	8.30%	12.40%	0.003^∗^	Class II > I

∗: significant (*p* < 0.05) in the Bonferroni post hoc test for chi-square tests.

## Data Availability

The data used to support the findings of this study are available from the corresponding author upon request.
